# Advancing a research agenda for bridging ageing and disability

**DOI:** 10.5334/ijic.1085

**Published:** 2012-11-16

**Authors:** Luis Salvador-Carulla, Michelle Putnam, Christine Bigby, Tamar Heller

**Affiliations:** Centre for Disability Research and Policy, Faculty of Health Sciences, The University of Sydney, Sydney, Australia; Simmons College School of Social Work, Boston, Massachusetts, USA; Research Leader Living with Disability, Faculty of Health Sciences, School of Allied Health, Department of Social Work and Social Policy, La Trobe University, Melbourne, Australia; Department of Disability and Human Development Director, Rehabilitation Research and Training Center on Aging with Developmental Disabilities, Director, Institute on Disability and Human Development, University of Illionis, Chicago, Illinois, USA

Research on ageing with disability dates back more than four decades. However, the evidence base supporting practice and policy across the fields of ageing and disabilities remains small in virtually all disciplines. In part, this can be attributed to the bifurcation of ageing and disability research, but it is also linked to the lack of a structural framework that supports bridging the areas of ageing and disability and the very few researchers working within these boundaries. In the past decade, declarations on the need to bridge ageing and disability have been forwarded [1–3], and the Toronto Declaration [4] now adds its voice from a global perspective. Despite the prior calls, advancement in bridging ageing and disability has been limited. The Toronto Declaration seeks to amplify the call for more integrative and interdisciplinary research and for effective knowledge transfer and translation of research into tangible outcomes for persons ageing with disabilities, their families, and their communities. In addition, it specifies the need for dedicated funding for this work and the inclusion of people with disabilities, of all ages, and their families in this work.

Research and knowledge transfer related to bridging ageing and disability involves complex challenges, engages very different stakeholders, including users, researchers, practice professionals, managers and policy-makers, and should apply novel models of care and research, as well as new concepts and techniques of analysis that require a substantial effort to be properly understood. Within this context this tentative research agenda is more illustrative than directive. It mentions fields that may be explored, some approaches that can be followed, and provides some key references to guide the reader in this quest.

## Advancing research

The Toronto Declaration delineates five priority areas for bridging ageing and disability knowledge, policy, and practice: 1) health and well-being, 2) inclusion, participation, and community, 3) long-term supports and services, 4) income security, and 5) the science of bridging. These areas are not discipline specific, but require engagement by research, practice, policy and consumer experts across domains of knowledge and experience. The Toronto Declaration [4] describes the global relevance of bridging knowledge in these areas and sets the overall purpose of bridging as “to improve efficiency, equity of care, inclusion and support at all levels, from the person to the society.” In many ways, these constitute goals for new research and knowledge transfer and translation efforts to which to contribute.

In broad terms, the practice, policy and research fields of ageing and disability have different approaches to understanding and addressing human functioning, disability, quality of life and well-being needs and issues. Historical origins of these different approaches vary across nations, but have direct impact on the international study of ageing and disability. Bridging the gap between ageing and disability theory continues to be a central challenge to advancing an ageing with disability research agenda but also for the transfer of existing knowledge across the ageing and disability sectors of scientific, practice and policy. This historical segmentation and its impact on research, practice and policy has been described elsewhere [5–8]. To the extent that research in ageing and disability continues to employ distinct models of understanding the experiences of ageing and living with disability, there is limited capacity to understanding shared issues, concerns, and factors that influence human functioning, disability, quality of life, and well-being or community outcomes. Additionally, there exist barriers to understanding the experiences, desires, and needs of the population of persons ageing with long-term disability and their families, who naturally span both categories.

Bickenbach et al. [9] discuss the important role the International Classification of Functioning (ICF) can play in helping to bridge ageing and disability theory. The ICF’s focus on identifying factors that mediate disability helps extend theoretical discourse into the realm of outcomes, embedding in some ways an implicit life course approach to reviewing disability over time, as well as a bio-psycho-social approach that takes into account the realms within which individuals and families exist. Other theoretical models that should be explored for their bridging potential might include models of social participation such as social inclusion [10, 11], the system-based functionality approach [12], and the life-span integrative approach, which incorporates a social capital framework [13]. The World Health Organization’s ‘active ageing’ framework should also be considered [14–16]. Work on the integration of ageing and disability theory will forward the study of ageing with disability and help create scaffolding for bridging ageing and disability sectors overall.

Production of evidence-based knowledge requires better integration of ageing and disability as well. Using the examples of integrated care, research analysis has mostly focused on the experimental approach of evidence-based care (EBC). However, in many cases the research behind evidence-based practices and policies is conducted on narrowly defined sample populations, which limits the transferability of findings across groups. Moreover, EBC studies are often conducted in controlled situations rather than in the more varied, real world community where most older people and those with disability of all ages live. This has significant implications for the development of new knowledge related to ageing with disability and for the transfer of existing evidence across ageing and disability practice fields. In social and health care, arguments have been made for moving from an exclusive evidence-based approach to an evidence-informed approach in relation to policy-making [17]. Realistic synthesis has been suggested as an alternative to classical EBC in the analysis of integrated care [18]. The evidence-informed approach applies evidence to priority setting and policy planning while taking into account observational and local data [19, 20] to determine what is best for a local community. As the knowledge base related to ageing with disability continues to develop, explorations of evidence-informed models of policy and practice may expand the potential of transferring knowledge between ageing and disability fields and identifying areas for additional research.

Models for how interdisciplinary bridging research can move forward may be found in areas that already hold common ground between ageing and disability sectors. Relevant to integrated care, and thus readers of this journal, is the area of person-centered care. Although interpretation of person-centered care varies between ageing and disability stakeholders, a general shared framework exists. The recently developed integrative matrix of positive and negative dimensions of specific care domains (i.e. health condition, human functioning, personal experience and determinants of health) [21] is one example where study of its application across age and disability groups provides an opportunity to bridge research sectors and engage in knowledge transfer activities. Another area where there is conceptual agreement across ageing and disability sectors is integrated care [22]. Although its implementation has been developed separately in each.

The Toronto Declaration [4] makes clear that based on the findings from the GOWD and FICCDAT meetings, inclusion of people with disabilities, of all ages, and their families must be “meaningfully included in bridging activities in recognition of their rights to self-determination and social inclusion.” Innovations and best practices for inclusion of direct stakeholders in bridging related research should be developed as part of a larger science of bridging.

## Knowledge transfer and translation

Bridging work and knowledge transfer in general, may be regarded as a ‘meta-science’ covering an array of different sectors, disciplines, approaches, and perspectives that require integration in order to produce evidence relevant to critical areas that generate progress in specific areas. Much of this science already exists. The Toronto Declaration [4] emphasizes the need to prioritize the transfer of knowledge across ageing and disability sectors to support national and international practice, policy, and research development. This work should be done in tandem to the development of new knowledge related to ageing with disability.

The science of knowledge transfer and translation is rapidly developing in response to calls for greater and faster application of research findings. Assessment of the utility of differing models of knowledge transfer and knowledge translation is important for bridging work related to ageing and disability. For example, Thigpen et al. [23] proposed a model of knowledge transfer and exchange emphasizing the rapid synthesis of existing literature and development of a product to guide practitioners as well as policy- decision-makers in the area of violence prevention. This model may hold particular relevance for bridging ageing and disability and for informing ageing with disability, as it seeks to quickly distribute known information and evidence to practice professionals and decision-makers while at the same time engaging a wide range of stakeholders in the work. Given that knowledge translations efforts tend to reflect the segmentation of ageing and disability, finding ways to embed bridging within knowledge transfer and translation efforts will be critical for creating strong bridging outcomes [24].

The science of bridging ageing and disability may also benefit from examining how other fields such as business and management design context-specific strategies that facilitate effective bridging and knowledge transfer. Use of business taxonomy [25] constitutes a paramount example of a system that effectively transfers knowledge across a broad sector. Applied to ageing and disability, a common health ontology including a glossary with standard definitions of key terms, a common semantic map, classification and hierarchy [26] will be important to facilitate the science of bridging ageing and disability. Equally important is an ontology of long-term care that allows for a common terminology, common instruments of assessment and a common classification of services [27]. In another example, it may be possible to learn from different fields such as Health Information Technologies (HiT) and Health Geography how to develop effective of regional, national, and international indicators of bridging activities to assess the outcomes of bridging work.

## Assessment of bridging

Assessment of bridging work will be critical for evaluating bridging strategies and outcomes and advancing research related to bridging ageing and disability. As part of this, developing an understanding of both successes and failures will be important. Internationally, significant national variations in research, practice, and policy related to ageing with disability exist. Comparative studies are needed to enhance understanding of the costs and benefits of bridging the two sectors and of the range of approaches in place to do so. The assessment of strategies that integrate older adults and younger persons with disabilities in health and social care programs has started to produce useful results in the US [28]. Understanding different national models and their utility has potential to more rapidly inform and advance policy supporting persons of all ages.

How can the effects of bridging be measured? Impact analysis techniques [29] and demonstration studies may provide useful answers. Also, new analytical techniques that take into account system dynamics, complexity, and prior expert knowledge such as Knowledge Discovery from Data [30] or Expert-based Cooperative Analysis [31] could be employed. Other models such as the WHO Systems Thinking model for strengthening health care systems may provide an excellent framework to analyse common delivery systems that serve older and younger persons with disabilities and their families [32].

## Funding for research and knowledge translation

Trends of global ageing for all people, including those with disabilities, indicates the work on building the science of bridging must begin in earnest. This will require research funding to support the development of new knowledge and to facilitate knowledge transfer and translation activities. Existing mechanisms supporting ageing and/or disability should more readily support work that bridges the two sectors. New mechanisms dedicated to supporting research on ageing with disability should be explored. Finally, there must be a strong commitment by the scientific community to support and engage in research that bridges ageing and disability and to acknowledge some flexibility in traditional and historical borders that segment ageing and disability.
